# Keap1/Nrf2 Signaling Pathway

**DOI:** 10.3390/antiox10060828

**Published:** 2021-05-22

**Authors:** Gerasimos P. Sykiotis

**Affiliations:** 1 Service of Endocrinology, Diabetology and Metabolism, Lausanne University Hospital, 1011 Lausanne, Switzerland; gerasimos.sykiotis@chuv.ch; Tel.: +41-21-3140-0606; 2 Faculty of Biology and Medicine, University of Lausanne, 1011 Lausanne, Switzerland

Nuclear factor, erythroid 2-like transcription factor 2 (Nrf2) and its cytoplasmic inhibitor, kelch-like ECH-associated protein 1 (Keap1), comprise a redox-responsive endogenous antioxidant defense module that orchestrates the expression of cytoprotective genes to maintain homeostasis [1]. Currently in its third decade, Keap1/Nrf2-related research continues to achieve landmarks, such as the recent demonstration of the role of Nrf2 in weight gain during space travel [2]. While much is already known about the regulation and functioning of the Keap1/Nrf2 pathway, basic research continues to reveal new insights; examples from the present Special Issue include distinct and overlapping roles in gene expression regulation with related cap’n’collar transcription factors Nrf1 and Nrf3 [3], as well as the connection of Nrf2 to cellular organelles with important emerging roles such as primary cilia [4]. In addition to general and ubiquitous antioxidant and other cell-protective genes, Nrf2 also regulates specific genes in each tissue that are critical for its functionality; for this reason, it has been coined “a multi-organ protector” [5]. Therefore, investigations of the protective effects of Keap1/Nrf2 signaling that focus on specific tissues or organs are of great interest. Such studies have traditionally focused on tissues with detoxification functions such as the liver [6,7] but have also expanded to diverse tissues, including the kidney [8,9], skin [10], lung [11], colon [12], heart [13], nervous system [14], adipose tissue [15], pancreas [16] and thyroid [17,18], as evidenced by the respective tissue/organ-specific studies that are outlined below and comprise the majority of studies in the present Special Issue. Lastly, research on Nrf2 has expanded to encompass not only basic but also translational and clinical studies. Clinical trials have indeed tested Nrf2 pathway modulators in the form of purified drugs or as dietary supplements for diverse indications such as cancer chemoprevention, detoxification of environmental pollutants, metabolic disease, diabetic nephropathy, relapsing forms of multiple sclerosis and others. Translational aspects addressed in this Special Issue include the epigenetic regulation of Keap1/Nrf2 signaling by phytochemicals [19], a patent review of the last 3 years [20] and, most notably, an overview of the current landscape of Nrf2 biomarkers in clinical trials [21]. The basic, tissue/organ-specific and translational studies in this Special Issue are briefly outlined below, starting from translational studies.

One the most impactful articles in this Special Issue is the review by Yagishita et al. on the current landscape of Nrf2 biomarkers in clinical trials [21]. They analyze results from clinical trials with four agents known to target Nrf2 signaling in preclinical studies (dimethyl fumarate, bardoxolone methyl, oltipraz and sulforaphane); for each of these agents, they evaluate the successes and limitations of six types of biomarkers: (i) Nrf2 target genes; (ii) gene expression/function; (iii) inflammation; (iv) oxidative stress; (v) carcinogen metabolism and adduct formation/clearance; and (vi) targeted and untargeted metabolomics. While the data indicate that each of these four lead clinical compounds does modulate the Nrf2 pathway in humans, utilization of the different types of biomarkers has been very unequal among the compounds. Another important limitation for clinical translation is that none of the biomarkers perform optimally at defining pharmacodynamic actions in the clinical setting [21]. This article is a must-read for anyone interested in pharmacological modulation of Nrf2 signaling, and it is expected to shape the field, not only in terms of utilizing the most suitable current Nrf2-related biomarkers for known and new compounds but also in terms of actively searching for better performing biomarkers to expedite clinical research.

Among the four compounds reviewed by Yagishita et al., sulforaphane is the most widely studied and the only one that has been associated with all six types of Nrf2 biomarkers [21]. Sulforaphane is also distinct from the other three compounds in that it is a natural substance, and specifically a phytochemical, isolated from various sources, notably broccoli [22]. In their article, Bhattacharjee and Dashwood review the current knowledge on epigenetic modulation of Keap1/Nrf2 signaling by phytochemicals, including sulforaphane and 16 other compounds. For each compound, they discuss the known effects on one or more of four types of epigenetic events: DNA methylation, histone modifications, epigenetic “readers” and regulation by non-coding RNAs. One of their major take-home messages is that none of these phytochemicals are likely to act by a single mechanism, or to affect a single molecular target without influencing other components of a signaling network [22]. This obviously complexifies both the interpretability of in vivo biomarkers and the prediction of safety due to potential side effects associated with off-target activities of the compounds.

Despite the current challenges in the clinical application of Keap1/Nrf2 signaling modulators, interest in realizing the translational potential of this pathway remains very intense. This is evidenced by the results of a patent search from my group by Chartoumpekis et al., covering the period 2017–2020 and focusing on patents mentioning Nrf2 [20]. More than 500 unique patents were identified that focused on various disease areas and referred primarily to activators of Nrf2 as potential treatments. A main theme among the topics of these patents was autoimmunity, covering various neuronal, gastrointestinal, endocrine and other diseases, associated with the well-established anti-inflammatory role of Nrf2. However, a key and surprising finding was that the experimental support for the respective patents’ claims was often insufficient, indicating that more research is warranted to support the potential beneficial effects of Nrf2 modulation in each respective autoimmune disease [20].

The majority of studies in this Special Issue report on the protective effects of Keap1/Nrf2 signaling in specific tissues or organs, which is related to the concept of Nrf2 as “a multi-organ protector” [5]. The most studied tissue in relationship to Nrf2 is the liver. The roles of Nrf2 in liver diseases are reviewed by Galicia-Moreno et al., covering molecular, pharmacological and epigenetic aspects [7]. One of their main conclusions is that various antioxidant compounds exert their effects by modulating Nrf2 signaling, yet few studies have elucidated the molecular modifications that these compounds exert specifically in the liver [7]. This further highlights the need for tissue-specific biomarkers of Nrf2 activity, as discussed above.

In an original research study, Chen et al. show that restraint stress, induced by limiting the ability of mice to move freely, alters expression of glucocorticoid bioavailability mediators, suppresses Nrf2 and promotes oxidative stress in the liver [6]. Specifically, they identified increased liver mRNA expression levels of the enzyme hydroxysteroid 11-beta dehydrogenase 1 (11β-Hsd1), which converts inactive glucocorticoids into active corticosterone, as well as decreased mRNA levels of 11β-Hsd2, which catalyzes the reverse reaction; this expression profile suggests an increased capacity for regeneration of active glucocorticoids in the liver. This was accompanied by oxidative and nitrosative imbalance and oxidative damage in the liver, as well as by decreased liver mRNA expression levels of Nrf2, even though classical Nrf2 target genes such as heme oxygenase 1 (Hmox1) and NAD(P)H quinone oxidoreductase 1 (Nqo1) were up-regulated, potentially via other mechanisms [6]. One limitation of this study, which is in fact shared with several other studies in the present Special Issue, is that Nrf2 knock-out mice were not employed to directly demonstrate an involvement of Nrf2 in the tissular and molecular phenotypes observed. Given that these mice are viable and fertile [23,24] (as are Nrf2 knock-out rats [25,26]), their use should be broadly encouraged in these types of investigations to move beyond obervational and correlative evidence and to facilitate mechanistic insights.

Besides the liver, the kidney is a major detoxification organ. Guerrero-Hue et al. review the protective roles of Nrf2 in renal disease [8]. This is a field of major clinical interest but also controversy, since a large phase 3 clinical trial of bardoxolone methyl in patients with chronic kidney disease and diabetes mellitus was terminated prematurely due to an increased risk of heart failure and cardiovascular events [27]. The underlying reasons for the observed toxicity remain unclear, and further clinical trials of bardoxolone methyl for renal diseases have been authorized. These aspects are discussed in detail in the comprehensive review by Guerrero-Hue et al. [8], and they are of relevance to anyone interested in clinical applications of Nrf2 activators, regardless of the indication.

In a preclinical research study in mice, Jiang et al. show that low-molecular weight peptides extracted from the head of a prawn (*Solenocera crassicornis*) have a protective effect against the renal toxicity of cyclophosphamide [9], which is used as a chemotherapeutic or immunosuppressive agent. Treatment of mice with the extract had beneficial effects on markers of inflammation, oxidative stress and apoptosis in the kidney, and this was accompanied by increased protein abundance of Nrf2 and increased mRNA expression levels of some of its target genes, including Hmox1 and Nqo1 [9]. However, like other studies, the data remain correlative because Nrf2 knock-out mice were not employed to demonstrate the mechanistic specificity and the magnitude of the Nrf2-attributable effect.

Beyond the main detoxification organs, Keap1/Nrf2 signaling has major roles in organs that come into contact with the external or internal environment, such as the skin, the lung and the colon. Ishitsuka et al. review the involvement of the Keap1/Nrf2 pathway in keratinization, the production of corneocytes that takes place in the skin and, more rarely, in other tissues [10]. Research in this field was stimulated by the observation that Keap1 knock-out mice die shortly after birth due to hyperkeratosis of the esophagus that blocks the passage of milk [28]. In their review, Ishitsuka et al. elegantly discuss the importance of Keap1/Nrf2 signaling and of a normal thiol (–SH) gradient through the epidermis for redox homeostasis in the skin; this provides a mechanistic basis to conceptualize the abnormal keratinization phenotypes observed in skin diseases associated either with oxidative stress or with strong and persistent Nrf2 activation [10].

In the lung, the Keap1/Nrf2 pathway has a central role not only in maintaining homeostasis and protecting against various diseases [29] but also in the aberrant activation of Keap1/Nrf2 signaling due to mutations or epigenetic modifications, which has been implicated as a driver event in lung carcinomas [30,31]. The study by Fabrizio et al. expanded on this knowledge by analyzing -omics data from a public repository corresponding to more than 1000 patients with non-small cell lung carcinoma (NSCLC) [11]. They report that the inverse correlation between the methylation levels of many CpG islands at various promoter and intragenic locations of the human *KEAP1* gene and the transcript levels were limited to patients with Kirsten rat sarcoma virus (KRAS) wild-type lung squamous cell carcinoma (LUSC) or lung adenocarcinoma (LUAD), whereas in LUAD, the effect of the epigenetic silencing of *KEAP1* on its transcription was also observed in patients with epidermal growth factor receptor (EGFR)-mutated tumors [11]. These results suggest that the prognostic role of such epigenetic alterations may differ among specific NSCLC histologies and molecular genetic backgrounds. The relevance of these findings for other cancer types with activation of Keap1/Nrf2 signaling also warrants exploration.

In a research study in mice, Li et al. show that dimethyl fumarate alleviates dextran sulfate sodium-induced colitis [12]. The phenotypic improvement is accompanied by up-regulation of the antioxidant enzymes glutamate-cysteine ligase catalytic subunit (Gclc) and glutathione peroxidase (Gpx), as well as by down-regulation of cyclooxygenase-2 (Cox-2) in colonic tissue. The authors postulate that these effects are mediated by the activation of Nrf2 [12]; however, like in other studies, this was not demonstrated directly by studying Nrf2 knock-out mice in parallel.

Reactive oxygen species are produced by mitochondria during activity in various muscle tissues, including the heart, where the role of Keap1/Nrf2 signaling in ischemia–reperfusion injury has been well established [32]. In a study using rats, Younis et al. extend these observations by showing that geraniol, an Nrf2-activating phytochemical, has protective effects on oxidative, inflammatory and apoptotic alterations in isoproterenol-induced myocardial infarction [13]. Similar to the aforementioned studies, involvement of Keap1/Nrf2 signaling is inferred indirectly by the induction of antioxidant and cytoprotective genes.

Neurons are particulary sensitive to oxidative stress, and they rely on their own antioxidant response as well as that of neighboring astrocytes to maintain homeostasis. In a review article, Kang summarizes recent studies on the relevance of Keap1/Nrf2 signaling for mitochondrial dynamics and/or mitophagy in various neurological diseases [14]. Based on a thorough analysis, it is concluded that a general pattern emerges wherein down-regulation of Nrf2 expression may be highly relevant for mitochondrial fragmentation in neurological diseases, but the precise profiles of the Nrf2 activity status and mitochondrial dynamics/mitophagy in each disease may be distinct [14].

Lastly, the roles of Keap1/Nrf2 signaling in endocrine tissues are also addressed in a set of studies in this Special Issue. In a study using mice, Cisterna et al. show that ex vivo treatment of explanted adipose tissue with ozone improves its preservation, which may be of relevance as an adjuvant treatment to improve graft survival in patients undergoing autologous fat transplantation [15]. This phenotypic improvement is accompanied by increased protein abundance of Nrf2 and increased mRNA expression levels of Hmox1; however, like in other studies, only wild-type animals were employed [15].

Another endocrine tissue that can be transplanted is the pancreas, and specifically the pancreatic islet cells for the treatment of diabetes. The outcomes of islet cell transplantation (ICT) depend, in large part, on the number and viability of the transplanted islet cells, which, however, are generally in short supply. Therefore, methods to improve the viability of islet cells for transplantation are actively pursued. Jarrin Lopez et al. review the results of recent studies that employed dimethyl fumarate or the Nrf2-activating phytochemical epigallocatechin gallate for ICT, and they discuss Keap1/Nrf2 signaling as a potential target to ameliorate oxidative stress at every step of the standard ICT protocol [16].

Finally, the thyroid gland has a special relationship with oxidative stress because it must not only defend itself against reactive oxygen species but it must also actively produce them as part of the thyroid hormone synthesis process. In a review article from my group, Thanas et al. discuss the latest studies on the roles of Keap1/Nrf2 signaling in the thyroid gland, which range from regulation of thyroid physiology to implication as a protective factor against diverse disease states including hypothyroidism, thyroid autoimmunity, thyroid enlargement (goiter), hyperthyroidism and thyroid carcinoma [18]. In addition, in an original research paper from my group, Chartoumpekis et al. describe the global gene expression pattern in the thyroid of mice in response to excess iodide; by studying wild-type and Nrf2 knock-out mice in parallel, it is conclusively shown that Nrf2 is critical for mounting protective antioxidant, anti-inflammatory/anti-autoimmune and anti-fibrosis transcriptional responses in this setting [17].

Even though preclinical research on Nrf2 is very active, and the first examples of clinical translation are already available, much remains to be learned about the basic mechanisms involved in the regulation and function of the Keap1/Nrf2 pathway. This is exemplified in the present Special Issue by two studies. The article by Ibrahim et al. employed RNA sequencing-based transcriptional profiling and quantitative proteomics to delineate the overlapping and differential genetic programs effected by Nrf2 and its closely related transcription factors Nrf1 and Nrf3 [3]. Although it has been known for several years that there are both common and dinstic target genes, for example, for Nrf1 and Nrf2 [33], recent studies using -omics approaches have begun to elucidate this issue at a more global level [34]. The article by Ibrahim et al. complements and extends prior knowledge by defining the consensus genetic targets of the Nrf family members from experiments performed with human cell lines [3]. An unexpected finding was that the basal expression of most Nrf1, Nrf2 and Nrf3 core consensus gene sets was not co-regulated across tissues, but the gene sets instead displayed tissue-specific preferences in their expression, such that genes co-regulated within one organ were often not correlated within another organ [3]. This indicates that caution should be exercised when gene expression levels are used as biomarkers of Nrf transcription factors, and that reliance on only one or two such biomarkers may give misleading results in specific tissues.

In another study, Martin-Hurtado et al. review the very novel topic of an emerging partnership between Nrf2 and primary cilia [4]. The primary cilium is a cellular organelle that forms in many cell types when not dividing as an antenna-like sensing and signaling structure around the centrosome, consisting of nine concentric microtubule pairs surrounded by a plasma membrane. Attesting to the important roles that primary cilia play in many tissues, their dysfunction has been associated with a diverse group of congenital human diseases termed ciliopathies, as well as with cancer [35]. As indicated by Martin-Hurtado et al., the fields of Nrf2 and primary cilia were completely separate as little as 5 years ago [4]. However, recent studies by their group and others have shown that a bidirectional relationship exists between cilia and Nrf2. Specifically, primary cilia can signal to Nrf2 via a primary cilium–autophagy–Nrf2 (PAN) axis [36]. Conversely, Nrf2 signaling can affect ciliogenesis, though there are contradictory results about whether it has a stimulatory effect [37] or an inhibitory one [38]. In their article, Martin-Hurtado et al. review the reciprocal functional interactions between primary cilia and Nrf2, discussing their possible pathophysiological implications and proposing possible explanations for the apparent contradiction regarding the effect of Nrf2 on ciliogenesis [4].

The collection of reviews and original research studies in this Special Issue illustrates the vibrant state of research into the various aspects of the Keap1/Nrf2 pathway, but also the persistent challenges in the translation of basic and preclinical research findings into new preventive or therapeutic strategies. At present, there are nine Special Issues in *Antioxidants* that are open for submissions with a direct or indirect focus on Nrf2, six of which have a clear translational/clinical focus (https://www.mdpi.com/journal/antioxidants/special_issues?section_id=0&search=nrf2&sort=deadline&view=open&page_count=50&query=nrf2; accessed on 18 April 2021). With such active research in the field, tangible further progress towards the elucidation of basic mechanisms, tissue/organ-specific functions and clinical translation can be credibly expected in the current decade.
